# Detection of Adverse Drug Reactions in COVID-19 Hospitalized Patients in Saudi Arabia: A Retrospective Study by ADR Prompt Indicators

**DOI:** 10.3390/healthcare11050660

**Published:** 2023-02-23

**Authors:** Ebtihal Al-Shareef, Lateef M. Khan, Mohammed Alsieni, Shahid Karim, Fatemah O. Kamel, Huda M. Alkreathy, Duaa A. Bafail, Ibrahim M. Ibrahim, Abdulhadi S. Burzangi, Mohammed A. Bazuhair

**Affiliations:** 1 Department of Pharmacology, Faculty of Medicine, King Abdulaziz University, Jeddah 21589, Saudi Arabia; 2 Department of Clinical Support Services, Pharmacy & Therapeutic Section, Royal Commission Medical Center, Yanbu 46451, Saudi Arabia

**Keywords:** adverse drug reactions, electronic medical record, prompt indicators, causality assessment tool

## Abstract

Seeking an alternative approach for detecting adverse drug reactions (ADRs) in coronavirus patients (COVID-19) and enhancing drug safety, a retrospective study of six months was conducted utilizing an electronic medical record (EMR) database to detect ADRs in hospitalized patients for COVID-19, using “ADR prompt indicators” (APIs). Consequently, confirmed ADRs were subjected to multifaceted analyses, such as demographic attribution, relationship with specific drugs and implication for organs and systems of the body, incidence rate, type, severity, and preventability of ADR. The incidence rate of ADRs is 37%, the predisposition of organs and systems to ADR is observed remarkably in the hepatobiliary and gastrointestinal systems at 41.8% vs. 36.2%, *p* < 0.0001, and the classes of drugs implicated in the ADRs are lopinavir-ritonavir 16.3%, antibiotics 24.1%, and hydroxychloroquine12.8%. Furthermore, the duration of hospitalization and polypharmacy are significantly higher in patients with ADRs at 14.13 ± 7.87 versus 9.55 ± 7.90, *p* < 0.001, and 9.74 ± 5.51 versus 6.98 ± 4.36, *p* < 0.0001, respectively. Comorbidities are detected in 42.5% of patients and 75.2%, of patients with DM, and HTN, displaying significant ADRs, *p*-value < 0.05. This is a symbolic study providing a comprehensive acquaintance of the importance of APIs in detecting hospitalized ADRs, revealing increased detection rates and robust assertive values with insignificant costs, incorporating the hospital EMR database, and enhancing transparency and time effectiveness.

## 1. Introduction

The world has experienced a massive devastating pandemic, creating extreme distress and a huge impact on all age groups, especially older persons with chronic disorders [1,2,3,4]. Remarkably, at the time of drafting this proposition, 631,935,687 infected individuals had led to a shocking number of deaths (6,588,850) across the globe according to the WHO dashboard on 14 November 2022 [5]. This global public health crisis was also reflected in the kingdom of Saudi Arabia (KSA) and, notably, there were 824,513 confirmed cases and 9433 deaths from January 2020 to 14 November 2022 [6]. This disastrous and highly contagious disease had gripped the entire world, with an absence of effective therapy to cease the upsurge and diminish morbidity and mortality [7]. The name coronavirus is derived from its unique morphology, as it comprises a solitary strand of positive-sense RNA (ribonucleic acid) and is highly contagious in nature, being transmitted mainly by the respiratory droplets of the infected person [8,9]. The basic essence of the pathogenesis of COVID-19 has a damaging effect on the lungs, but severely infected patients may develop dysfunction of the kidney, liver, cardiovascular, neurological, and hematological systems. However, the majority of the complications are essentially due to the ‘cytokine storm’ [9]. The hallmark of this ‘cytokine storm’ leads to the development of multiorgan failure and, logically, becomes the chief cause of prolonged hospitalization [9]. Remarkably, coronavirus causes clinical manifestations when replicating, hence the impacting antiviral therapy will be more effective if used before the illness extends to the active inflammatory phase proceeding to the cytokine storm [7,10].

However, selecting antiviral drugs and managing COVID-19 patients presents a great challenge and creates a therapeutic dilemma for clinicians. Paradoxically, the antiviral agents used for optimizing the treatment of COVID-19 patients are empirical, including remdesivir, chloroquine, hydroxychloroquine, lopinavir/ritonavir, and favipiravir, etc. [10,11]. This markedly, required the need for immediate and effective therapies, leading researchers to use repurposing approaches, permitting multiple benefits, such as reducing risk, time, and costs. In addition, it provides easy access to data regarding the pharmacokinetics, pharmacodynamics, and ADRs of the concerned drugs [12,13]. Thus, utilizing such approaches diminishes the time required to explore an effective treatment for COVID-19, reducing morbidity, mortality, and long-term consequences [14,15,16,17].

It is vital to ensure appropriate drug safety whenever seeking enhanced efficiency [18]. However, confronting a pandemic of such a great magnitude with no definitive treatment, the incidence of ADRs in patients with COVID-19 is bound to be unpredictable. Moreover, at present, a standard method for the detection of ADRs is still imperfect and relies almost entirely on a spontaneous reporting system [19,20], suffering heavily from its inherent limitations [21,22]; hence, suitable alternative methods are required for the detection of the ADRs. 

A comprehensive pharmacovigilance method has been devised to detect ADRs by utilizing APIs [20,22]. The basic essence of this approach is to resolve the dilemma of under-reporting and excessive time consumption, and apparently, it has a positive impact on authentic drug safety and rational use of drugs. The primary objective of this research is to identify and characterize the pattern of ADRs due to COVID-19 drugs, such as demographic features, the incidence, and types of ADR, causality assessment, duration of hospitalization, number of drugs taken in the hospital, history of past drug allergies, history of chronic diseases; and the secondary objective is observing the out-come of drugs used in COVID-19 patients, such as the most common drugs implicated in ADRs, the system and organs involved in ADRs, preventability, and severity of ADRs, thereby facilitating and enhancing drug safety. 

## 2. Methodology

### 2.1. Study Design and Population

This retrospective study plans to accomplish the detection and analysis of ADRs from the EMR database by selected APIs, using the active monitoring model of the National Coordinating Council for Medication Error Reporting and Prevention Index (NCC MERP Index) [23], illustrating medication harm and comprising: Class E: Temporary harm to the patient and required intervention; Class F: Temporary harm to the patient, requiring initial or prolonged hospitalization; Class G: Permanent patient harm; Class H: Intervention required sustaining life; and Class I: Patient death. The duration of this study was 6 months from April to October 2021. The current study was conducted by enrolling 381 patients with a confirmed diagnosis of COVID-19. The study was approved by the ethical approval committee of the institute, with permission to seek the records from the inpatients’ EMR database. Because of the retrospective nature of the study, written informed consent is not required. 

### 2.2. Selection of “ADR Prompt Indicators”

A professional group, an expert team consisting of two clinical pharmacologists and one medical internist, agreed upon and approved the list of “APIs” based on the signals employed in past studies [22,24] and on an identical study on the use of trigger tools [25]. The EMR database was thoroughly checked to detect and analyze the ADRs. It must be emphasized that APIs used in the current study identify the adverse event harmful to the patient and excludes medication errors. 

Inclusion criteria: Patients included in this study are of either sex, aged more than 18 years, with a confirmed diagnosis of COVID-19, and are recipients of at least one medication and/or positive laboratory indicator of the APIs (Table 1).

Exclusion criteria: patients not provided with any treatment, transferred to another department or discharged within 48 h of admission. 

### 2.3. Active Data Collection and Surveillance

The basic characteristics of the selected patients with COVID-19, i.e., age, sex, nationality, admission details, length of hospital stay, drug intake, use of antiviral drugs, history of drug allergies, and chronic diseases, were extracted from the EMR database of the hospital. Subsequently, the entire drug therapy of all included patients were systematically analyzed to recognize the triggers or the “prompt indicators”, in order to identify the presence of suspected ADRs, by the team of reviewers (comprising two clinical pharmacologists and one medical internist), systematically scrutinizing the information, establishing the occurrence of ADR, and utilizing two causality assessment tools, i.e., clinical judgment and Naranjo’s algorithm tool [26].

### 2.4. Causality Assessment of ADRs

During the current study, the potential ADR detection process was exclusively based on the notion that whenever the “API” is recognized from the record of the database, it is only considered as a suggestive “positive indicator” and not an “established ADR.” Subsequently, these suspected ADRs are confirmed as ADRs by the aforesaid assessment tools [26]. Finally, the association between the recognized ADRs and the drugs is marked as definite, probable, and possible for inclusion in the study. 

### 2.5. Pilot Study

To establish the suitability of the designed study to reveal the ADRs of COVID-19 drug therapy, a pilot study was initially accomplished from the information of 50 patients acquired from the EMR database.

### 2.6. Comprehensive Correlation between ADRs and Characteristics of the Patients

Consequently, the demographic characteristics of the patients extracted from the EMR database were further analyzed for specific organs and systems implicated in ADR, precise drug therapy and drug intake, suspicious drugs and their involvement in the ADR, clinical outcome, type of ADR, until eventually the incidence rate of ADRs is determined. Ultimately, the clinical consequences of ADRs, comprising cure (after the symptoms of ADR have disappeared) or recovery of the abnormal indicators, is recorded appropriately.

### 2.7. Determination of Preventable ADRs

Moreover, the rationality of any drug therapy with eventual augmentation of drug safety is only made possible by the determination of preventable ADRs; our study has achieved this precisely by utilizing the Hartwig scale [27]. 

### 2.8. Identification of the Severity of ADRs

Finally, suitable intervention to stimulate the pharmacovigilance, and assessment of the severity of ADRs is considered to be crucial in any study for reinforcing the rationality of drug therapy; this vital facet was fulfilled in the current study by the method of Shumock and Thornton [28].

### 2.9. Statistical Analysis

The descriptive and analytical data was analyzed by using SPSS 20 software package, performing univariate, and multivariate analysis to determine the relations of prospective risk factors with hazard regarding ADRs, expressing the result suitably in percentage and absolute numbers and testing statistical significance, either by 95% confidence intervals or *p*-value < 0.05, interpreted as statistically significant. 

## 3. Results

### 3.1. Demographic Characteristics of Patients

The number of patients in current study comprises 381 patients, of whom 78 are women and 303 are men. The mean age of the patients is 48.93 ± 14.63. Patients with a history of chronic diseases are 42.5%. Remarkably, API’s initial finding for the suspected ADRs is positive in 176 patients. However, on further analysis, 35 ADRs are observed to be disease-related symptoms and marked as “false positive”, hence discarded. Finally, 141 patients are categorized with confirmed ADRs, whereas patients without ADRs are 240, thereby demonstrating the incidence rate of ADRs as 37%.

**Figure 1:**
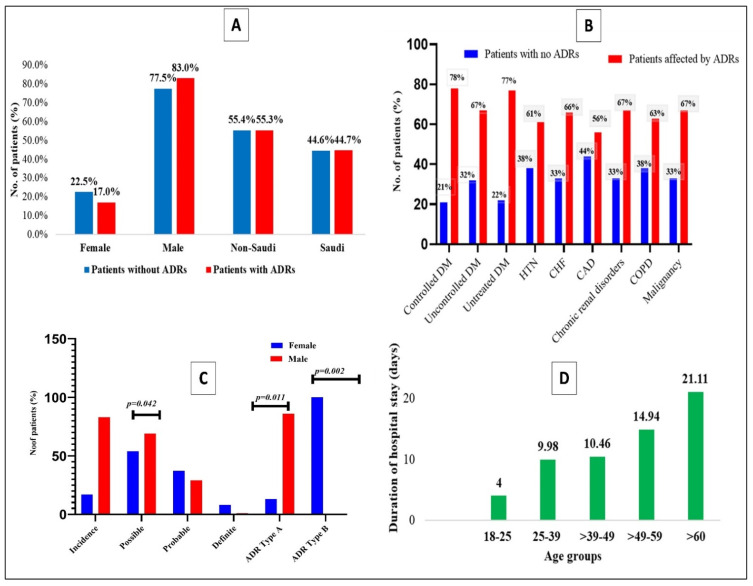
(**A**) Demographic features of the patients influenced ADRs and unaffected by the ADRs, (**B**) Causality, incidence, and type of ADRs in COVID-19 patients, (**C**) Relationship of ADRs in COVID-19 patients with different comorbidities, (**D**) Relationship of ADRs between different age groups and duration of their hospital stay. *p*-value < 0.05 is considered as significant.

Especially, on comparing the demographic characteristics of patients, between those with and without ADRs (Table 2 and Figure 1A), there are insignificant changes in patients without and with ADRs regarding age (48.46 ± 14.74 versus 49.74 ± 14.45, *p* = 0.409) and gender (female 54 (22.2%) versus 24 (17.0%) and males (186 (77.5%) versus 117 (83.0%), *p* = 0.125), whereas non- Saudi patients are more often without ADRs than with ADRs (55.4% versus 55.3%, *p* = 0.024). Age, gender, and nationality were not risk factors for ADRs due to infection with COVID–19 (*p* = 0.408, *p* = 0.202, and *p* = 0.985, respectively).

### 3.2. Clinical Characteristics of Patients of Those with and without ADRs

It needs to be highlighted that the duration of hospitalization and the number of drugs taken by the patients are observed to be significantly higher in patients with ADRs in comparison to those without ADRs (14.13 ± 7.87 versus 9.55 ± 7.90, *p* < 0.001 and 9.74 ± 5.51 versus 6.98 ± 4.36, *p* < 0.0001) (Table 3 and Figure 1D). Nevertheless, combined antiviral drug intake is higher in patients without ADRs than in those with ADRs (73.3% versus 53.2%, *p* < 0.0001). The risk factors for patients with ADRs are prolonged hospital stay (RR: 1.038; 95%CI: 1.014–1.063; *p* < 0.002) and intake of several drugs in the hospital (RR: 1.127; 95%CI: 1.075–1.182; *p* < 0.0001), in addition to the usage of a single antiviral agent (RR: 2.420; 95%CI: 1.563–3.748; *p* < 0.0001). Besides this, there are no significant changes when comparing patients with and without ADRs regarding the history of past drug allergies (Table 3).

### 3.3. Influence of Comorbidities over the Development of ADRs among the COVID-19 Patients

The current study describes the essential emphasis while observing the influence of different comorbidities on patients with COVID-19 (Table 4 and Figure 1) Markedly, 162/381 (42.5%) patients have different comorbidities and, remarkably, 106 (75.2%) patients have developed different ADRs, while additional evaluation has revealed both controlled/uncontrolled DM, HTN and CHF displaying significant ADRs (*p*-value < 0.05).

### 3.4. Identification of ADRs Using Medications and Laboratory Prompt Indicators

It needs to be emphasized that, predominantly, ADRs are gastrointestinal tract (GIT) disorders (36.2%), detected by medication prompt indicators (MPIs); concomitantly 2.8% of skin ADRs are also recognized by MPIs. On the contrary, merely 3.5% of electrolyte ADRs are revealed with MPIs (Table 5), 41.8% of the hepatobiliary ADRs are detected by laboratory prompt indicators (LPIs), while 10.68% of hyper-lipidemic disorder ADRs are detected by LPIs (Table 5).

### 3.5. Causality, Incidence, and Type of Adverse Drug Reactions

The incidence of ADRs in the current study (Table 6 and Figure 1C) is revealed as 37%, with a higher rate of ADRs in males in comparison to females. Besides, in causality assessment, males show a higher possibility of ADRs compared to females (69.2% versus 54%, *p* = 0.042). In contrast, type A ADRs are detected more in males than females. Surprisingly, ADR Type B is found exclusively in females (*n* = 4; 100%) (Table 6 and Figure 1C). However, the causality of ADRs displayed the pattern of possible > probable > definite.

### 3.6. Adverse Drug Reaction (ADR) with Regards to Age in the Current Study

The propensity of ADRs is observed to be more prevalent in males and noticeable in the age groups 49–59 and >60 years. Furthermore, this predominance of ADRs is generalized more in males among entirely all age groups. Besides, their duration of hospitalization is also found to increase proportionately according to the increase in age group (Table 7). There is a significant and coherent relationship between age, increase in hospitalization and ADRs (*p* = 0.0001 vs. *p* = 0.001), respectively.

### 3.7. The Organs and Systems Involved in Patients with ADRs

Emphatically, the most frequent ADRs are observed to be associated with hepatobiliary, gastrointestinal (GIT), and hyperlipidemic disorders (41.8% vs. 36.2% and 10.6%, respectively). Moreover, GIT-related ADRs 36.2% are more frequently attributed to antiviral agents (14.9%), followed by antimalarial drugs (9.2%), then antibiotics (8.5%). On the other hand, hepatobiliary ADRs are also attributed principally to antiviral agents (23.4%), then antibiotics (10.6%), and thereafter monoclonal antibodies (3.6%). As regards the hyperlipidemic disorders, corticosteroids are exclusively responsible for all the ADRs labeled as hypercholesteremia while hypertriglyceridemia results from monoclonal antibodies (2.13%), followed by antiviral agents at 1.42%, and corticosteroids at 0.71% (Table 8).

### 3.8. Potentiality of the Most Common Drugs Involved in ADRs of COVID-19 Patients

It is worth mentioning that the interesting fact revealed by the result of our study is that most of the ADRs are detected with antibiotics 34 (24.1%) and antiviral drugs 58 (41.1%). However, lopinavir/ritonavir produces 16.3% of ADRs, which in turn culminates in four (2.8%) serious adverse drug reactions (SADRs); additionally, two (1.4%) SADRs are observed with favipiravir and oseltamivir, and one with Tocilizumab (0.7%). Regarding causality assessment, probable ADRs are 43 (30.4%), possibly 94 (66.6%) and definite 4 (2.8%) out of the 141 ADRs observed. Nevertheless, we have observed that all the encountered ADRs are tackled effectively by symptomatic management, after which 70.2% fully recovered and 29.8% of the affected patients were cured, Given the establishment of the correlation between the SADRs and the drugs /organs implicated, our results illustrated that 5.7% are induced by antiviral and immunosuppressant agents (Lopinavir/ritonavir, and Favipiravir and Tocilizumab), which manifested as an acute liver injury with conclusively full recovery (Table 9).

### 3.9. Association of ADRs and Medications Used for COVID-19

In the current study, antiviral medications are found to be the most common drugs responsible for ADRs and account for 58 /141 (41.1%) (Table 10). From these ADRs, the maximum number of corresponding to hepatobiliary disorders was 23.4%, and to GIT disorders 14.9%. This is followed by antibiotics, contributing to 34/141 (24.1%) ADRs, relating to hepatobiliary disorders (10.6%) and GIT disorders (8.51%).

### 3.10. Preventability and Severity of ADRs

**Figure 2:**
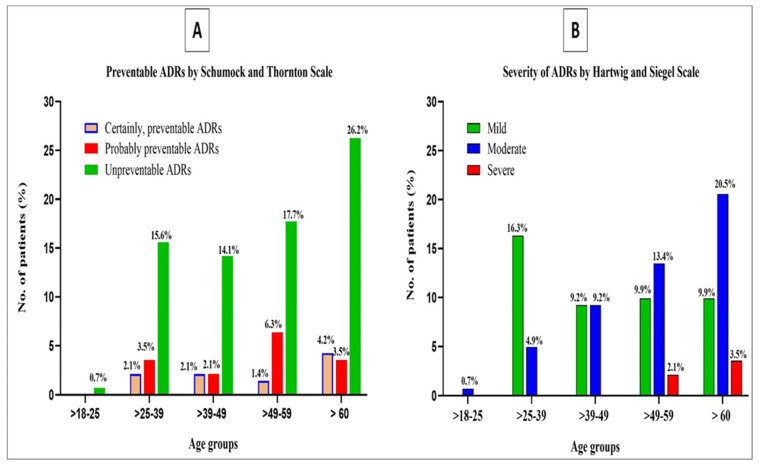
Relationship of preventability and severity of ADRs corresponding to the different age groups observed in hospitalized patients with COVID-19. (**A**) Preventable ADRs (**B**) Severity of ADRs.

In the current study, certainly preventable ADRs are identified and documented in different age groups as 9.9%, and probably preventable ADRs in all ages as 15.6%. It needs to be emphasized that unpreventable ADRs are more frequently observed in the age groups of 49–60 and >60 years, where the likelihood of associated chronic health disorders is more pronounced. However, the figure for non-preventable ADRs is exhibited as 74.5% (Table 11 and Figure 2). Nevertheless, the elementary core and precise tagging of certainly preventable ADRs with specific attribution correlates to hepatobiliary disorders at 8.51%; surprisingly, all the GIT disorders related to ADRs account for 48.6%, revealed as non-preventable (Table 11 and Figure 2). 

The current study highlights and demarcates the severity of ADRs and, on the scale of severity, we observed 64 (45.4%), 69 (48.9%) and 8 (5.7%) as mild, moderate and severe, respectively (Table 11). Furthermore, the severe category of ADRs is detected in the age groups of 49–59 and <60 years, designated as a high-risk factor for chronic health care disorders, eventually augmenting susceptibility to the incidence of ADRs. Moreover, looking closely at severe ADRs, it is observed that 3.5% of severe ADRs attribute to hematological disorders and 2.1% to hepatobiliary disorders (Table 11 and Figure 2). Conversely, substantial numbers of mild ADRs are observed with GIT disorders at 36.2%, and the majority with moderate ADRs at 41.8%, accredited to hepatobiliary disorders (Table 11). 

## 4. Discussion

The precipitous and devastating global outbreak of COVID-19 raising a dilemma internationally, spreading at a rapid velocity. This substantially reinforced the prospect of a huge impact on community health and therefore necessitated decisive efforts to explore the trends of epidemiology, which in turn helped in becoming more alert in encountering the high prevalence of COVID-19. Our current study incorporates the utilization of APIs to recognize ADRs, this monitoring system enhancing the frequency of ADR reporting and significant diminution of ADR under-reporting with regard to drug safety concerns of patients with COVID-19 [18,29]. 

This retrospective study describes the demographic characteristics of hospitalized COVID-19 patients in Yanbu, KSA. Fundamentally, the hallmark of any pharmaco-epidemiological study is to ascertain the incidence rate of ADRs; this vital component plays a crucial and unequivocal task in regulating the quality of health care of patients by recognizing those methods diminishing morbidity and mortality [30,31] In the current scenario of the COVID-19 pandemic, rapid infectivity and lack of definitive antimicrobial agents greatly worsened this quality indicator, shaking public trust in the present healthcare system [32,33]. The current results display a total of 141 (37%) ADRs among 381 patients, and several identical recent studies conducted in Saudi Arabia, Malaysia, and China for the detection of hospitalized ADR using ADR trigger tools have also illustrated an escalated incidence rate of ADRs, which varies from 22.8% to 74.2%, [34,35,36]. This strikingly alarming healthcare parameter is a serious concern for the improvement of both safety and the overall quality of care provided to hospitalized patients. It needs to be emphasized that, whenever the incidence rate of a disease process is disturbingly increased, there is the likelihood of an enormous decline in public trust in the governing healthcare system, therefore requiring adequate safety measures [30].

### 4.1. Characteristics of the Patients

The present study reported 20.5% women and 79.5% men among the 381 patients included, indicating a higher incidence of susceptibility in the male; correspondingly, ADRs are more common in males at 83.0% than in females at 17%; however, identical observations are also illustrated in recent studies [37,38,39]. The high preponderance of male gender susceptibility to COVID-19 in comparison to females is attributed to several factors, such as differentials in inherent immunity and sex hormones. Identifying compelling evidence for the vital role executed by the androgen in the coupling of transmembrane serine pprotease2 (TMPRSS2) in alliance with the angiotensin-converting enzyme 2(ACE2) gene on the X-chromosome triggering augmented ACE2 levels may perhaps lead to a favorable effect in females infected with COVID-19 [39]. Interestingly, identical phenomena regarding the demographic dominance of male gender susceptibility over females to COVID-19 hospitalization are reported in almost all the provincial regions of Saudi Arabia [36]. In contrast, less likelihood of female infectivity with COVID-19 has been attributed to the disparity in innate immunity, steroid hormones, and additional influences, coupled with the sex chromosomes [40]. Nevertheless, lifestyle factors, such as less smoking and less drinking of alcohol, along with the attitude of females, more sensitive towards this pandemic, this might have a positive effect by leading to more precautions, such as wearing a face mask, frequent hand washing, and staying at home [41]. 

### 4.2. Relationship of Age to the Development of ADRs

The present study portrays the ADRs in the age group of 39–49 years as 18.4% while vulnerability in the elderly age >60 contributed to 34% of the ADRs (Table 7). In addition, the severity of ADRs in the current study is reported as 5.67%. (Table 11) Thus, the contribution of adults seems to affect the highest proportion of ADRs in COVID-19 patients. This conforms with earlier studies [42,43]. The similarity of the current study’s results in this regard is reflected in a large database study from 17 million patients in Britain. In contrast, more than 90% of infected children are either asymptomatic or have mild to moderate disease [44]. The reasons accountable for this important contributory factor to the severity of ADRs in COVID-19 in adult patients, as well as increased mortality due to COVID-19 infections due to age, include alterations in the production of T and B lymphocytes, worsening in lung capacity, and atrophic changes in bronchial smooth muscles contributing to a decline in lung reserves and the clearance of airways. Furthermore, enhanced coagulopathy and acute myocardial and liver injury are the most frequent complication noted after COVID-19 infections in the geriatric age group [45,46]. 

### 4.3. Drug-Induced ADRs in Hospitalized Patients of COVID-19 Are Directly Proportional to the Total Amount of Drug Intake and Simultaneously Prolonged Hospitalization

A remarkable, obvious, and inevitable core feature of hospitalized ADRs in patients of COVID-19 in this study is the effect of ADRs on the duration of hospital stay and concurrently the effect of the intake of several drugs on ADRs (Table 3). Both these dual components are significantly (*p* < 0.001) higher in patients with ADRs than in those without ADRs. Many similar phenomena are reported in other studies [47,48,49,50]. The variety of predisposing factors escalating the risk of developing hospitalized ADRs includes prolonged hospitalization, polypharmacy, comorbidities, inappropriate medication use and cardiovascular disorders. [50]. Furthermore, a systematic review illustrated that the presence of multiple chronic disorders and polypharmacy are among the top ten hazardous factors for ADRs [50,51]. Thus, the period of a patient’s hospitalization is ultimately detrimental to health care costs [18].

### 4.4. The Impact of Comorbidities on the Development of ADRs in COVID-19 Patients

It is clear that patients with intrinsic uncontrolled chronic healthcare disorders, such as diabetes mellitus, hypertension, chronic vital organ disorders and cancer, are highly susceptible to acquiring COVID-19 infection, more complications and ADRs [52,53]. Furthermore, recent studies have revealed that the outcome seems to be more severe in individuals with comorbidities when infected with COVID-19 in comparison with patients with no comorbidities [53,54]. The results of the current study reciprocate and highlight the statistically identical phenomena revealed in the aforesaid latest studies [52,53,54]. Hence, it is important to raise a global public campaign to develop awareness in order to lessen the liability of morbidity and mortality in this situation [54,55,56].

### 4.5. Relationship of Medication and Laboratory Prompt Indicators in Hospitalized ADRs Due to COVID-19

The prime objective of the utilization of medications and laboratory prompt indicators in the current study for the fundamental detection of ADRs is effectively accomplished, by utilizing the well-recognized ADR trigger tools that have a discrete and intrinsic significance of a higher detection rate and lesser cost compared to the conventional methods [20]. In addition, they also have the distinct benefit of recognizing preventable ADRs in comparison to the traditional methods, while a spontaneous reporting system is often accompanied by significant under-reporting [36]. In the current study, ADRs related to the GIT in hospitalized patients were significantly detected collectively by the MPI: Metoclopramide, Ondansetron and Loperamide at 36.2% (*p* < 0.0001), in contrast to 43.5% and 20.5% in recent similar types of studies [34,36]. However, LPI for detecting liver injury accounted for 41.8% of ADRs (*p* < 0.0001) in the current study, in comparison to 36.5% and 36.2% in the aforesaid recent studies [34,36]. At this juncture, focusing on this issue, the current data from the literature revealed that major medications responsible for provoking acute liver injury in COVID-19 patients are antimalarial and antimicrobial [57], and perhaps ACE2 and hypoxic damage to the liver are the most conceivable mechanism for the development of these ADRs [58,59,60]. Surprisingly, apart from the present drugs used, other possible mechanisms attributed to acute liver injury in this scenario could be concomitant drug therapy, underlying disease of the liver, and the viral effect itself [61]. 

### 4.6. Causality Assessment of ADRs in the Current Study

The basic hallmark and the likelihood of the causality assessment are to identify and distinguish an ADR as “definite” or “suspected”, with the prime objective to transmute skepticism to irrevocability [62].This process is essential, yet it is a complicated process in pharmacovigilance to establish the correlation between the suspected ADR and the usage of a specific drug. However, it is self-evident that the detection of ADRs makes a significant contribution towards early recognition, preventing relapse in sufferers of ADRs, thus optimizing drug therapy with the ultimate aim of enhancing the quality of care of the patients [63]. In the current study, ADRs are assessed by using the Naranjo scale [23] and 30.5% of these reactions were classified as probable, 66.6% as possible (*p* < 0.042) and only 2.8% as “definite,” due to the complexity of evaluating a causal relationship between a drug and an adverse reaction. It has been highlighted that, currently, several similar studies are being conducted around the globe with the identical objective of the current study, but demonstrating distinct observations regarding their results relating to causality assessment [35,64,65,66]. This can be explained based on the adopting of numerous methodologies and different settings leading to ambiguities regarding the causal link between the ADRs and the underlying disease associated with the confounding factors, contending with the drug as possible cause of the ADRs [57].

### 4.7. Organs and Systems Involved in Hospitalized ADR in COVID-19 Patients

There seems to be a reciprocal and inadvertent relationship between the drugs used in hospitalized patients with COVID-19 and the increased susceptibility of their organs and systems to ADRs, conceivably due to weaker and suppressed body defense mechanisms, this scenario in turn perpetuating the augmented use of multiple medications and anticipation of potential drug–drug interaction [61]. According to the current study, hepatobiliary, digestive system and hyperlipidemic disorders are most affected by ADRs, according to the results of Sun et al. [35]. Analogous characteristics are observed in several recent studies of COVID-19 [33,35,36,67,68,69,70]. As a repercussion of the aforesaid observations, it appears to be coherent that hepatobiliary ADRs seem to be multifactorial and heterogenous in nature, which could be due to direct viral binding to ACE2-positive cholangiocytes, and this can cause hepatic injury, in addition to activation of the immune system and “Cytokine Strom”, promoting immune-mediated hepatic injury. It is pertinent to point out that altered liver function, disease severity and older age contribute to half of the incidence of ADRs in COVID-19 infections [71,72,73,74].

### 4.8. Potentiality of the Most Common Drugs Involved in ADRs of Patients Treated for COVID-19

By far, ADRs are seemingly the exclusive and crucial affiliate for weak adherence to treatment, hence it is always essential to initially optimize the choice of the antiviral agent before commencing therapy in hospitalized COVID-19 patients; regrettably, this could not be accomplished due to the unique and sudden onset of this pandemic. However, the stark reality observed in the current study revealed that the most reported class of drugs suspected of causing ADRs in COVID-19 hospitalized patients are antiviral (41.13%), antibiotics (24.1%) and antimalarial drugs (12.8%), and the most frequently related drugs for the reported ADRs are lopinavir/ritonavir (16.3%), antibiotics (24.1%) and hydroxychloroquine (12.8%). The current study’s results are consistent with numerous recent analogous studies [35,36,57,64,67,68,75]. Sun et al. 2020 [35] have illustrated in their study that the proportionality of the ADRs produced by lopinavir/ritonavir (63.8%) seems to be the maximum. In contrast, in the study of Yang et al., 2020 [75], ADRs associated with lopinavir/ritonavir were 33.5%, but this disparity could be due to the modest sample size of his study. Furthermore, another recent study that was conducted by Ramírez et al. 2020 [1] in a tertiary care hospital in Spain testified in a remarkable observation that in the COVID-19 patients the incidence rate of serious ADRs is observed to be 4.75 times greater than that perceived in non-COVID-19 patients [75]. Strikingly, an enthusiastic and comprehensible element of information is revealed by Chouchana et al. (2021) [76], comparing the therapeutic drug monitoring of lopinavir-ritonavir levels in the serum of 24 COVID-19 patients with patients infected with human immunodeficiency virus, in which it is observed that lopinavir plasma concentrations rise by 4.6–8-fold in COVID-19 patients and this exorbitant rise of serum levels specifically necessitates care in circumventing the ADRs [77]. It needs to be highlighted that almost all the observed ADRs, including the grievous category, are confronted efficiently by symptomatic management, and no mortality was revealed in the current study.

### 4.9. Relation between ADRs and Medications Used for Treatment of COVID-19

The fundamental hallmark of this contemporary study is that the therapeutic remedies accountable for the ADR give a candid reflection of most similar studies in Saudi Arabia and worldwide. Illustrating our current finding of ADRs (65.2% with *p*-value < 0.0001) due to antimicrobial involvement, it is noticeable that several public sector tertiary care hospitals in Lahore, Pakistan, conducted prospective cross-sectional observational studies with an identical ADR incidence rate of approximately 38.9% attributed to antimicrobial agents [77], and strikingly this also corresponds to studies conducted in Saudi Arabia [21,78]. The most realistic and noteworthy triggering factor and justification for this multiplicity of ADRs related to antimicrobial agents is the high prevalence of their use. Indeed, antimicrobials are the most consistently prescribed medications in Saudi Arabia [79].

### 4.10. Preventability and Severity of ADRs

The current study is highly determined to account for the evaluation of preventable and severe ADRs linked to hospitalized COVID-19 patients. The purpose of detecting preventable ADRs in any epidemiological study is to reinforce the rationality of drug therapy, which in turn augments drug safety [18,22]. A consistent focus in any epidemiological study of ADRs is the strong possibility of its preventability, which is outwardly decisive for strengthening the rationality of drug therapy in order to enhance drug safety [20]. ADRs determined by the Schumock and Thornton Scale [28] are observed to be preventable in 25.5% of all age groups and remarkably associated with liver and biliary system disorders (Table 11 and Figure 2). However, non-preventable ADRs are detected at 74.5%, (Table 11 and Figure 2), mostly in the age group of >50 years, and most were ascribed as GIT disorders (Table 11). It must be reiterated that ADRs can be prevented by staying away from medications that have previously caused an ADR, avoiding medications that are inappropriate for the patient, optimizing the dosage regimen, performing regular monitoring tests, and checking for significant drug interactions. These are the most important interventions and the findings of recent studies in Saudi Arabia and our current study [36,77]. 

In addition, the current study reveals that the severity of ADRs (mild = 45.4% and moderate = 48.9%) is demonstrated chiefly in the age group of >50 years (Figure 2). In addition, current analogous studies illustrated a comparable proportion for the severity of ADRs [36,64]. The crucial feature of drug safety is moreover reinforced by evaluating the severity of ADRs to assist in the identification of the vital facets of intervention in order to strengthen drug safety. Seemingly, this assertion is satisfactorily accomplished in the current study, enlightening the focus of interest in the perusal of the severity of ADRs in this age group and highlighting and substantiating a high-risk factor for chronic health care disorders, which in turn augments the susceptibility to the incidence of ADRs. As a ramification of the aforesaid observations, it is explicit that evaluation of these fundamental aspects of ADRs is a prerequisite of pharmacovigilance and would take up a pivotal position in reducing the burden of ADRs, reducing healthcare costs, and ultimately boosting quality care of the patient [18]. 

The results of our current study demonstrated an escalated incidence rate of ADRs at 37%, which distinctly matches several identical recent studies of hospitalized patients of COVID-19, conducted in Saudi Arabia and across the globe, with identical methods using trigger tools. Moreover, it is noteworthy that in this study the propensity of males over females to be susceptible to ADRs also closely resembles the finding of studies throughout the world. Furthermore, the current results in terms of susceptibility based on the vulnerability of age and length of hospital stay also correspond with several current studies. Ultimately, this study reiterates and reinforces the perception that APIs illustrate the most robust method to evaluate the EMR database for the detection of ADRs.

## 5. Limitation of the Study

Our study has utilized APIs for the detection of ADRs, often criticized for their inherent limitation of being biased and providing false positives, but this was effectively surmounted by the robust expert team of two clinical pharmacologists and one medical internist, who thoroughly scrutinized the information from the EMR database to confirm the ADRs, utilizing two causality assessment tools. Additionally, the current study detected ADRs exclusively in hospitalized patients, hence was unable to predict the comprehensive ADRs of all COVID-19 patients, focusing only on one center for Yanbu residents, hence its outcomes cannot be applied to the entire Saudi population. 

## 6. Conclusions

Certainly, this study contributes to illustrating the fundamental parameters of ADRs related to the drugs utilized against COVID-19 and appears to be symbolic in providing a comprehensive acquaintance of the importance of APIs as the state of the art in recognizing in detecting hospitalized ADRs in patients with COVID-19. Moreover, it revealed escalated detection rates and robust assertive values with concomitant lesser cost, their incorporation into the hospital EMR database leading to further augmentation of transparency and time effectiveness. 

## Figures and Tables

**Table 1:** List of APIs employed for monitoring ADRs in hospitalized patients of COVID-19.

ADR Prompt Indicators	
**Laboratory indicators**	
Platelet count < 3.5 × 10^0^/L	Drug-influenced platelet diminution
Serum ALT, AST, T Bilirubin or ALP > 2ULN	Drug-influenced liver injury
Serum cholesterol > 6 mmol/L	Drug-influenced hypercholesterolemia
Serum Triglyceride > 1.7 mmol/L	Drug-influenced hyperlipidemia
**Medication indicators**	
Dextrose 50%	Hypoglycemia
Metoclopramide and Ondansetron	Drug-influenced nausea and vomiting
Promethazine and chlorpheniramine	Skin allergy
Phytonadione	Drug-influenced coagulation disorder
Sodium polystyrene and Calcium polystyrene	Drug-influenced Hyperkalemia
Loperamide	Drug-influenced diarrhea

**Table 2:** Demographic features of patients influenced by ADRs and unaffected by ADRs.

Variables	All Patients (*n* = 381)	Patients without ADRs (*n* = 240)	Patients with ADRs (*n* = 141)	*p*-Value	Univariate Analysis	
					Relative Risk (95% CI)	*p*-Value
Age	48.93 ± 14.63	48.46 ± 14.74	49.74 ± 14.45	0.409	1.006 (0.992–1.020)	*p* = 0.408
Gender				0.125		
Female	78 (20.5%)	54 (22.5%)	24 (17.0%)		* A	
Male	303 (79.5%)	186 (77.5%)	117 (83.0%)		1.415 (0.830–2.413)	*p* = 0.202
Nationality				0.024		
Non-Saudi	211 (55.4%)	133 (55.4%)	78 (55.3%)		* B	
Saudi	170 (44.5%)	107 (44.6%)	63 (44.7%)		1.004 (0.661–1.525)	*p* = 0.985

* *p*-value < 0.05 is considered as significant. * A = Female is the standard and Male is compared to Female. * B = non-Saudi is the standard and Saudi is compared to non-Saudi.

**Table 3:** Clinical features of the patients influenced by ADRs and unaffected by ADRs.

Variables	Total Patients (*n* = 381)	Patients with No ADRs (*n* = 240)	Patients Affected by ADRs (*n* = 141)	*p*-Value	Univariate Analysis	
					Relative Risk (95% CI)	*p*-Value
Duration of hospital stay	11.25 ± 12.7	9.55 ± 7.90	14.13 ± 7.87	0.001	1.038 (1.014–1.063)	*p =* 0.002
Quantity of drug intake	8.00 ± 4.99	6.98 ± 4.36	9.74 ± 5.51	0.0001	1.127 (1.075–1.182)	*p* < 0.0001
Use of antiviral agent				0.0001		
Combined use of antiviral	251 (65.9%)	176 (73.3%)	75 (53.2%)		* C	
Single-use of antiviral	130 (34.1%)	64 (26.7%)	66 (46.8%)		2.420 (1.563–3.748)	*p* < 0.0001
History of past drug allergies				0.507		
No	274 (71.9%)	173 (72.1%)	101 (71.6%)		* D	
Yes	107 (28.1%)	67 (27.9%)	40 (28.4%)		1.023 (0.644–1.623)	*p* = 0.924
History of chronic diseases				0.117		
No	219 (57.5%)	184 (76.7%)	35 (24.8%)		* E	
Yes	162 (42.5%)	56 (23.3%)	106 (75.2%)		1.320 (0.868–2.008)	*p* = 0.195

*p*-value < 0.05 is considered as significant. * C = Multiple antiviral agents are the standard and single antiviral compared to multiple antivirals, * D = Patients without past drug allergies are standard, and patients with past drug allergies are compared to patients without past drug allergies * E = Patients without a history of chronic disease are the standard and patients with chronic disease are compared to patients without chronic disease.

**Table 4:** Relationship of ADRs in COVID-19 patients with different comorbidities.

COVID-19 Patients with Comorbidities	Total Patients (*n* = 381)	Patients with No ADRs (*n* = 240)	Patients Affected by ADRs (*n* = 141)	Univariate Analysis	
				Relative Risk (95% CI)	*p*-Value
Without comorbidity	219 (57.5%)	184 (76.7%)	35 (24.8%)	* E	
With comorbidity	162 (42.5%)	56 (23.3%)	106 (75.2%)	1.320 (0.868–2.008)	0.195
Controlled DM	14	3 (21.4%)	11(78.6%)	6.685 (1.832–24.391)	0.001
Uncontrolled DM §	37	12 (32.4%)	25 (67.7%)	4.095 (1.986–8.444)	0.001
Untreated DM §	9	2 (22.2%)	7 (77.8%)	3.511 (0.864–14.266)	0.062
HTN	54	21 (38.9%)	33 (61.1%)	0.295 (0.140–0.625)	0.001
CHF §	12	4 (33.3%)	8 (66.7%)	3.549 (1.049–12.007)	0.031
CAD §	16	7 (43.7%)	9 (56.3%)	2.269 (0.826–6.234)	0.103
Chronic renal disorder	09	3 (33.3%)	6 (66.7%)	3.511 (0.864–14.266)	0.062
COPD§	8	3 (37.5%)	5 (62.5%)	2.904 (0.683–12.342)	0.131
Malignancy	03	1 (33.3%)	2 (66.7%)	3.439 (0.309–38.271)	0.285

*p*-value < 0.05 is considered significant. * E = Patients without a history of comorbidities are the standard for patients with comorbidities compared to those without comorbidities. **§** Congestive heart failure (CHF), coronary artery disease (CAD), Chronic obstructive pulmonary disease (COPD), diabetes mellitus (DM), hypertension (HTN).

**Table 5:** Confirmed ADRs detected by the Medication and Laboratory prompt indicators selected for the study.

Medication Prompt Indicators (MPI)	MPI Confirmed as ADRs (*n* = 62; 43.9%)	*p*-Value	Lab Prompt Indicators (LPI)	LPI Confirmed as ADRs (*n* = 79; 56.1%)	*p*-Value
Dextrose 50%	-	-	Platelet count < 120 PLT/μL	5 (3.5%)	*p* < 0.0001
Promethazine	-	-	Serum ALT > 55 U/L	21 (14.89%)	*p* < 0.0001
Chlorpheniramine	4 (2.8%)	*p* < 0.0001	Serum AST > 34 U/L	28 (19.9%)	*p* < 0.0001
Metoclopramide	23 (16.3%)	*p* < 0.0001	Serum T Bilirubin > 20.5 μmol/L	5 (3.5%)	*p* < 0.0001
Phytonadione	2 (1.4%)	*p* < 0.0001	Serum ALP > 150 U/L	5 (3.5%)	*p* < 0.0001
Sodium polystyrene	-	-	Serum Cholesterol > 6 mmol/L	9 (6.38%)	*p* < 0.0001
Calcium polystyrene	5 (3.5%)	*p* < 0.0001	Serum Triglyceride > 2.4 mmol/L	6 (4.3%)	*p* < 0.0001
Loperamide	8 (5.7%)	*p* < 0.0001			
Ondansetron	20 (14.2%)	*p* < 0.0001			

*p*-value < 0.05 is considered significant.

**Table 6:** Causality, incidence, and type of ADRs in patients in the current retrospective study.

Character	Total (*n* = 381)	Female (*n* = 78)	Male *(n* = 303)	*p*-Value
Incidence	141 (37.0%)	24 (17%)	117 (83%)	0.188
Definite	04 (2.8%)	02 (8.3%)	02 (1.7%)	0.187
Probable	43 (30.5%)	9 (37.5%)	34 (29%)	0.536
Possible	94 (66.66%)	13 (54%)	81 (69.2%)	0.042
ADR Type A	137 (97.1%)	19 (13.8%)	118 (86.1%)	0.011
ADR Type B	04 (2.8%)	04 (100%)	-	0.002

*p*-value < 0.05 is considered significant. The causality assessment according to the Naranjo scale.

**Table 7:** Correlation among ADRs corresponding to age groups and the duration of hospital stay of hospitalized patients with COVID-19.

Age Groups	Total (*n* = 141)	Female (*n* = 24) 17.1%	Male *(n* = 117) 82.9%	*p*-Value	Duration of Hospital Stay (Days) (Mean ± SD)	*p*-Value
18–25 (*n* = 1)	1	1 (4.2%)	-	0.0001	4.00 ± 0.00	0.001
25–39 (*n* = 30)	30	3 (12.5%)	27 (23.1%)		9.98 ± 5.82	
39–49 (*n* = 26)	26	4 (16.7%)	22 (18.8%)		10.46 ± 4.98	
49–59 (*n* = 36)	36	7 (29.2%)	29 (24.8%)		14.94 ± 7.99	
>60 (*n* = 48)	48	9 (37.5%)	39 (33.3%)		21.11 ± 8.50	

*p*-value < 0.05 is considered as significant.

**Table 8:** Organs and the systems implicated in ADRs of hospitalized patients with COVID-19.

Organ and the Systems Involved	Medication Groups & Their Intake	No of the ADRs (*n* = 141)	Incidence of ADRs (%)	*p*-Value
Skin reactions (*n* = 4)	Antibiotics (*n* = 34)		2.1%	0.044
	Antiviral (*n* = 58)	1	0.7%	0.454
GIT disorders (*n* = 51)	Antibiotics (*n* = 34)	12	8.5%	0.537
	Antiviral (*n* = 58)	21	14.9%	0.567
	Antimalarials (*n* = 18)	13	9.2%	0.001
	Monoclonal antibodies (*n* = 11)	2	1.4%	0.168
	Other drugs (*n* = 10)	3	2.1%	0.48
Hepatobiliary disorders (*n* = 59)	Antibiotics (*n* = 34)	15	10.6%	0.454
	Antiviral (*n* = 11)	33	23.4%	0.002
	Antimalarials (*n* = 10)	4	2.84%	0.058
	Monoclonal antibodies (*n* = 11)	5	3.6%	0.52
	Other drugs (*n* = 10)	2	1.4%	0.130
Hyperkalemia (*n* = 5)	Antiviral (*n* = 58)	1	0.7%	0.314
	Other drugs (*n* = 10)	4	2.8%	0.0001
Hypercholesterolemia (*n* = 9)	Corticosteroids (*n* = 10)	9	6.4%	0.0001
Hypertriglyceridemia (*n* = 6)	Corticosteroids (*n* = 10)	1	0.7%	0.362
	Antiviral (*n* = 58)	2	1.4%	0.52
	Monoclonal antibodies (*n* = 2)	3	2.1%	0.006
Thrombocytopenia (*n* = 5)	Antibiotics (*n* = 34)	3	2.1%	0.091
	Antimalarials (*n* = 18)	1	0.7%	0.5
	Monoclonal antibodies (*n* = 11)	1	0.71%	0.338
Bleeding disorder (*n* = 2)	Other drugs (*n* = 10)	2	1.4%	0.005

*p*-value < 0.05 is considered as significant.

**Table 9:** Potentiality of drugs to develop ADRs, their causality evaluation, SADRs, and treatment outcome in patients with COVID-19.

Potential Drugs to Produce ADRs	All ADRs (*n* = 141) 100%	Serious ADRs (*n* = 8) 5.7%	Definite (*n* = 4) 2.8%	Probable (*n* = 43) 30.4%	Possible (*n* = 94) 66.6%	Outcome	
						Cure (*n* = 99) 70.2%	Recovery (*n* = 42) 29.8%
Lopinavir/ritonavir	23 (16.3%)	4 (2.8%)	1 (0.7%)	10 (7.1%)	12(8.5%)	16 (11.4%)	7 (4.9%)
Favipiravir	14 (9.9%)	2 (1.4%)	1 (0.7%)	5 (3.5%)	8 (5.67%)	9 (6.3%)	5 (3.5%)
Oseltamivir	21 (14.9%)	1(0.7%)	-	3 (2.1%)	18 (12.77%)	13 (9.2%)	8 (5.6%)
Hydroxychloroquine	18 (12.8%)		-	6 (4.2%)	12 (8.51%)	11(7.8%)	7 (5.0%)
Tocilizumab	11 (7.8%)	1 (0.7%)	2 (1.4%)	4 (2.8%)	5 (3.55%)	9 (6.4%)	2 (1.4%)
Corticosteroids	10 (7.1%)		-	7 (4.9%)	3 (2.13%)	6 (4.3%)	4 (2.84%)
Antibiotics	34 (24.1%)		-	7 (4.9%)	27 (19.2%)	27 (19.2%)	7 (5.0%)
Medications used for chronic disorders	10 (7.1%)	-	-	1 (0.7%)	9 (6.4%)	8 (5.7%)	2 (1.4%)
Total	141 (100%)	8 (5.7%)	4 (2.8%)	43 (30.4%)	94 (66.6%)	99 (70.2%)	42 (29.8%)

*p*-value < 0.05 is considered significant.

**Table 10:** Correlation between ADRs and medications used for the treatment of COVID-19.

Class of Drug in ADR *n* (%)	Skin ADR *n* (%) 4 (2.8)	GIT ADR *n* (%) 51 (36.2)	Hepatobiliary Disorder *n* (%) 59 (41.8)	Hyperkalemia *n* (%) 5 (3.5)	Hypertriglyceridemia *n* (%) 6 (4.2)	Hypercholesterolemia *n* (%) 9 (6.4)	Thrombocytopenia *n* (%) 5 (3.5)	Bleeding Disorder *n* (%) 2 (1.4)	*p*-Value
Antibiotics *n* = 34 (24.1%)	3 (2.1%)	12 (8.5%)	15 (10.6%)	-	-	-	3 (2.1%)	-	*p* < 0.0001
Corticosteroids *n* = 10 (7.1%)	-	-	-	-	1 (0.7%)	9 (6.4%)	-	-	*p* < 0.0001
Antiviral *n* = 58 (41.1%)	1 (0.7%)	21 (14.9%)	33 (23.4%)	1 (0.7%)	2 (1.4%)	-	-	-	*p* < 0.0001
Antimalarials *n* = 18 (12.8%)	-	13 (9.2%)	4 (2.8%)	-	-	-	1 (0.7%)		*p* < 0.0001
Monoclonal antibodies *n* = 11 (7.8%)	-	2 (1.4%)	5 (3.6%)	-	3 (2.13%)	-	1 (0.7%)	-	*p* > 0.050
Chronic disorder medications *n* = 10 (7.1%)		3 (2.1%)	2 (1.4%)	4 (2.8%)	-	-	-	2 (1.4%)	*p* > 0.050

*p*-value < 0.05 is considered significant.

**Table 11:** Preventability and Severity of ADRs regarding the organ and systems involved in hospitalized patients of COVID-19.

The System Involved	Schumock and Thornton Scale of Preventable ADRs			Hartwig and SIEGEL Scale of Severity of ADRs		
ADRs *n* = 141 (%)	Certainly, Preventable ADRs 14 (9.9%)	Probably Preventable ADRs 22 (15.6%)	Unpreventable ADRs 105 (74.5%)	Mild 64 (45.4%)	Moderate 69 (48.9%)	Severe 8 (5.7%)
Skin reactions 4 (2.8%)	-	-	4 (2.8%)	-	4 (2.8%)	-
Gastrointestinal disorders 51 (36.2%)	-	-	51 (48.6%)	51 (36.2%)		-
Hepatobiliary disorders 59 (41.8%)	12 (8.51%)	6 (4.3%)	41 (29.1%)	10 (7.1%)	46 (32.6%)	3 (2.1%)
Electrolyte disorders (Hyperkalemia) 5 (3.5%)	-	3 (2.1%)	2(1.4%)	2 (1.4%)	3 (2.1%)	-
Hyperlipidemic disorders (Hypercholesterolemia 9 (6.4%) & Hypertriglyceridemia 6 (4.2%))	-	13 (9.2%)	2 (1.4%)	1 (0.7%)	14 (9.9%)	-
Hematological disorders (Thrombocytopenia 5 (4.3%) & Bleeding disorder 2 (1.4%))	2 (1.4%)	-	5 (3.55%)	-	2 (1.4%)	5 (3.5%)

## Data Availability

The datasets used and/or analyzed during the present study are available from corresponding author on reasonable request.

## Abbreviations

ADR prompt indicators (APIs), adverse drug reactions (ADRs), angiotensin-converting enzyme 2 (ACE2), Chronic obstructive pulmonary disease (COPD), congestive heart failure (CHF), coronary artery disease (CAD), coronavirus disease 2019 (COVID-19), diabetes mellitus (DM), electronic medical record (EMR), gastrointestinal tract (GIT), hypertension (HTN), Institute for Healthcare Improvement (IHI), the kingdom of Saudi Arabia (KSA), lab prompt indicators (LPI), medication prompt indicators (MPI), National Coordinating Council for Medication Error Reporting and Prevention (NCC MERP), ribonucleic acid (RNA), serious Adverse drug reactions (SADR), severe acute respiratory syndrome coronavirus-2 (SARSCoV-2), transmembrane serine protease 2 (TMPRSS2), World Health Organization (WHO).
